# Greater glucose uptake heterogeneity in knee muscles of old compared to young men during isometric contractions detected by [^18^F]-FDG PET/CT

**DOI:** 10.3389/fphys.2014.00198

**Published:** 2014-05-28

**Authors:** Thorsten Rudroff, John H. Kindred, John-Michael Benson, Brian L. Tracy, Kari K. Kalliokoski

**Affiliations:** ^1^Integrative Neurophysiology Laboratory, Department of Health and Exercise Science, Colorado State UniversityFort Collins, CO, USA; ^2^Turku PET Centre, University of Turku and Turku University HospitalTurku, Finland

**Keywords:** positron emission tomography, glucose, aging, muscle volume, computed tomography

## Abstract

We used positron emission tomography/computed tomography (PET/CT) and [^18^F]-FDG to test the hypothesis that glucose uptake (GU) heterogeneity in skeletal muscles as a measure of heterogeneity in muscle activity is greater in old than young men when they perform isometric contractions. Six young (26 ± 6 years) and six old (77 ± 6 years) men performed two types of submaximal isometric contractions that required either force or position control. [^18^F]-FDG was injected during the task and PET/CT scans were performed immediately after the task. Within-muscle heterogeneity of knee muscles was determined by calculating the coefficient of variation (CV) of GU in PET image voxels within the muscles of interest. The average GU heterogeneity (mean ± SD) for knee extensors and flexors was greater for the old (35.3 ± 3.3%) than the young (28.6 ± 2.4%) (*P* = 0.006). Muscle volume of the knee extensors were greater for the young compared to the old men (1016 ± 163 vs. 598 ± 70 cm^3^, *P* = 0.004). In a multiple regression model, knee extensor muscle volume was a predictor (partial *r* = −0.87; *P* = 0.001) of GU heterogeneity for old men (*R*^2^ = 0.78; *P* < 0.001), and MVC force predicted GU heterogeneity for young men (partial *r* = −0.95, *P* < 0.001). The findings demonstrate that GU is more spatially variable for old than young men and especially so for old men who exhibit greater muscle atrophy.

## Introduction

To accomplishtypical daily tasks, adequate muscle activation and force are necessary but this function may be impaired in elderly. Age-related alterations in neuromuscular activation may contribute to the impairments; however, the underlying physiological mechanisms in the aging neuromuscular system have yet to be fully understood. Recruitment of motor units and modulation of firing rates of active motor units are the two mechanisms available to the nervous system for regulation of muscle force. Previous studies investigated motor unit discharge characteristics in order to clarify the age related changes in the neuromuscular system (Nelson et al., 1983; Ross et al., 1997, 1999; Kamen, 2005; Pascoe et al., 2013). However, this information is provided from protocols that used intramuscular electromyography (EMG). Although this technique can provide important information about spinal motor neuron behavior, there are several limitations. These limitations include: representation of only a small portion of the muscle, invasiveness, and only a limited number of muscles can be examined. More recently, multichannel surface EMG has been used to estimate motor unit behavior during force production (Farina et al., 2008; Merletti et al., 2008) and findings have demonstrated that spatial activation in a muscle is non-uniform and that spatial EMG potential distribution pattern is altered by contraction levels and fatigue. The heterogeneity of the location of different muscle fiber types and a clustering of muscle fibers innervated by one motor neuron in a limited region of the muscle might explain this phenomenon (Holtermann et al., 2005, 2008; Holtermann and Roeleveld, 2006). Watanabe et al. (2012) used multichannel EMG to compare spatial potential distribution from the vastus lateralis muscle between old and young men while they performed ramp isometric knee extension from 0 to 65% of maximal voluntary contraction (MVC). They showed that the old men were not able to activate the working muscle homogeneously during contractions.

Previous studies have shown that modulation of motor unit activity during voluntary sustained contractions varies with the characteristics of the load against which the limb acts. When young and old subjects performed submaximal isometric contractions with the lower leg muscles, for example, the adjustments in motor unit activity to sustain the same net muscle torque differed when the ankle pulled against a rigid restraint to match a target force (force task) compared with maintaining a constant joint angle while supporting a more compliant load (position task) (Griffith et al., 2010). The average (a) EMG activity and EMG bursting activity of agonist and antagonist muscles was greater during the position task due compared with the force task in young and old adults. Furthermore, Hunter et al. (2005) reported similar EMG findings and also briefer times to failure for the position task when old adults performed the tasks with the elbow flexor muscles. However, although surface EMG is a useful measure of muscle activation, there are limitations to the information that can be extracted from the signal (Farina et al., 2004). For example, as indicated by a previous study (Rudroff et al., 2013), surface EMG is sometimes not able to detect differences in muscle activation across tasks. Furthermore, surface EMG cannot provide information about heterogeneity of activation deep in muscle and among deep and adjoining muscles.

Positron emission tomography (PET) with [^18^F]-fluoro-deoxy-glucose ([^18^F]-FDG) as a glucose analog (tracer) provides detailed information about muscle activation (Fujimoto et al., 1996, 2000, 2003; Pappas et al., 2001; Kemppainen et al., 2002) because of the non-insulin dependent uptake in working muscles. PET can also provide a measurement of heterogeneity within the tissue (coefficient of variation) which has been used to investigate muscle blood flow heterogeneity and the underlying mechanisms (Heinonen et al., 2010, 2013; Rudroff et al., 2014), also during isometric contractions (Kalliokoski et al., 2003). With PET method, glucose uptake (GU) is measured within small 3D volume elements (voxels). Thus, a muscle is divided into small samples and the heterogeneity is calculated as the coefficient of variation (SD/mean) of these GU values of voxels within each muscle (Kalliokoski et al., 2000; Heinonen et al., 2012). Apparently, as the tracer is accumulated over a long period of time, the measure of heterogeneity represents solely spatial heterogeneity and temporal variation in GU within the voxels cannot be determined.

Heinonen et al. (2012) showed that GUh provides an estimate of activation of muscle fibers within the muscles. As more motor units, and thus more muscle fibers, are activated during fatiguing contractions, GUh is expected to decrease. However, no previous studies have investigated GUh during sustained submaximal isometric contractions in young and old men. In the current study we used a voxel by voxel analysis to determine spatial parameters based on data collected in the previous study (Rudroff et al., 2013). This allows us to determine whether GU heterogeneity differs between young and old men and thus may provide an indicator of more heterogeneous muscle activation strategies employed by old men.

The purpose of the study was to estimate spatial heterogeneity of [^18^F]-FDG uptake within skeletal muscles when young and old men performed isometric contractions with the knee extensors that required either force or position control. We expected that old men will have greater heterogeneity of skeletal muscle GU during two types of contractions than young men due to their inability to activate the working muscle homogeneously. Furthermore, we hypothesized that GUh will be more pronounced in the position task for young and old men.

The findings add to the outcomes of our previous study (Rudroff et al., 2013) indicating that young and old use different muscle activation strategies to accomplish force and position tasks.

## Methods

Participants' characteristics, physical activity levels, study design, and PET imaging acquisition have been previously reported in Rudroff et al. (2013).

### Subjects

Six young (26 ± 6 years) and six old (77 ± 6 years) men with similar body mass (young men: 77.3 ± 5.9 kg; old men: 79.0 ± 6.2 kg, *P* = 0.7) participated in the three separate experimental sessions that comprised the study protocol. Informed consent was obtained from all participants, who reported being free from cardiovascular and neurological disorders and participating in moderate levels of structured physical activity (2–4×/week). The experimental procedures were approved by the Institutional Review Board at the University of Colorado Boulder and were in accordance with the *Declaration of Helsinki*.

### Physical activity levels

To obtain an estimate of habitual physical activity level, the participants wore an accelerometer (ActiGraph GT3X, Pensacola, FL) mounted at the hip to record accelerations in the vertical and horizontal directions during waking hours for 7 consecutive days. The accelerometers recorded data in 60-s intervals and also counted the number of steps. The data were downloaded onto a Microsoft Excel spreadsheet using the ActiLife software. Data recorded on the first and last days were discarded and only data sets for at least 4 complete days (including 1 weekend day) were used in the comparison, consistent with current recommendations (Mâsse et al., 2005).

### Study design

Tasks were performed at each session. Before each task, the subject performed several MVC trials with the left knee extensor muscles. The MVC task comprised a 3-s increase in force from zero to maximum with the maximal force held for ~3 s, and subjects were verbally encouraged to achieve maximal force. Subjects rested for 60–90 s between trials. When the peak forces achieved in two of the three trials differed by >5%, additional MVCs were performed until this criterion was met. The greatest force achieved by each subject was taken as the MVC force. This initial MVC was used to determine the target force for the subsequent contraction. All tasks required the subject to sustain a single leg (left) isometric contraction with the knee extensors at 25% of the MVC force. At this target force, blood flow is impaired but not occluded (Sadamoto et al., 1983). The first visit involved determining the endurance time for the position task with the target force set at 25% MVC. It has been shown that the position task is more difficult and the time to failure is briefer than for the force task (Enoka et al., 2011). The other two sessions, which included either the force or position task, were performed on separate occasions, with 1 week between the sessions, in a randomized order, and in a room adjacent to the PET/CT scanner. The subjects accomplished the tasks for 90% of endurance time for the position task as determined in the first session. During the force task, the left knee extensors of the subjects pulled against a rigid restraint and matched the force exerted by the leg to the target force that was displayed on a monitor (1% MVC/cm vertical displacement on display). The other fatiguing contraction, position task, required subjects to use the knee extensors to support an equivalent inertial load and to maintain the position of the leg by matching knee angle to the target displayed on the monitor (1°/cm vertical displacement on display).

Immediately after the target time was achieved, the subject was placed in the PET/CT scanner to measure [^18^F]-FDG uptake in selected muscles. PET/CT scans were preformed twice for each subject, after the force task and after the position task.

The subject performed all tasks in a supine posture to limit the influence of accessory muscles, especially those in the upper body. The trunk-thigh angle was at 3.14 rad, the left knee joint angle at 0.78 rad, and the right knee angle at 1.57 rad. A strap was placed around the waist to stabilize the subject and another strap was wrapped around the ankle to connect the load to the leg. The force exerted by the leg was measured with a load cell (0–500 lb, Noraxon, Scottsdale, AZ) placed in series with the load during the position task. The force signal was low-pass filtered (5 Hz) and recorded on a computer (1000 samples/s). The cable connecting the ankle to the rigid restraint for the force task was adjustable to achieve the desired knee joint angle. Knee joint angle during the position task was measured with a flexible 2D goniometer sensor (Noraxon, Scottsdale, AZ) secured to the lateral aspect of the knee joint. The output of the goniometer was recorded, displayed on a monitor, and stored (1000 samples/s) on a computer. The inertial load (25% MVC force) for the position task was suspended from the ankle at the same location that the restraint was applied during the force task. The force task was terminated when the subject was not able to achieve the target force for 5 s, and the position task was ended when the subject was unable to maintain the knee angle within 0.17 rad of the target value. Immediately after each task subjects performed another MVC, which was used to derive an index of fatigability.

### PET imaging

Prior to the two sessions in which PET/CT imaging was to be performed, the subjects were required to fast for at least 4 h, refrain from any kind of strenuous activity for at least 1 day, and to consume water and void the bladder just before the experiment. After the MVC had been determined, a buffalo cap line was placed into the antecubital vein of the right arm to deliver the tracer. A finger stick was used before the injection of [^18^F]-FDG to determine the level of plasma glucose concentration. Approximately 2 min after the start of the fatiguing contraction, ~7.2 mCi of [^18^F]-FDG in 5 ml of saline was infused into the vein and the sustained contractions continued thereafter for 729 ± 137 s (young men) and 631 ± 83 s (old men). Immediately after the injection of the tracer, the buffalo cap was removed. Once the fatiguing contraction had been sustained for the prescribed duration (90% of endurance time for the position task), the subject was moved into the scanner within 2 min and the acquisition and processing of the PET/CT images was performed following the standard protocol used in the Division of Nuclear Medicine, Department of Radiology, University of Colorado School of Medicine, Denver, CO.

The PET scans were performed with a GE Discovery ST scanner (General Electric Medical Systems, Milwaukee, WI, USA). The scanner has 24 PET detector rings of Bismuth Germinate (BGO) crystals forming 47 two-dimensional imaging planes with a sampling interval of 3.27 mm each. The lower limb was scanned from hip to feet in short CT scans for attenuation correction and 2-min emission scan time frames, with 6–7 frames for each subject depending on the height of the individual. Both sets of data were acquired consecutively one frame at the time with the subject on the same scanning table and in the same position. The feet and lower legs of the subject were secured to maintain co-registration. Total PET/CT scan time was ~28 min. The data sets were reconstructed using an iterative method (OLSEM) with 21 subsets and 2 iterations with a Gaussian filter into 355 transaxial slices (each 4 mm thick). Voxel size in each slice was 4 × 4 mm and thus, the final #d voxels size was 4 × 4 × 4 mm. All data sets were corrected for dead-time and random coincidence. The axial and in-plane resolution of the reconstructed images was ~5 mm full-width at half maximum.

### Imaging analysis

**Figure 1:**
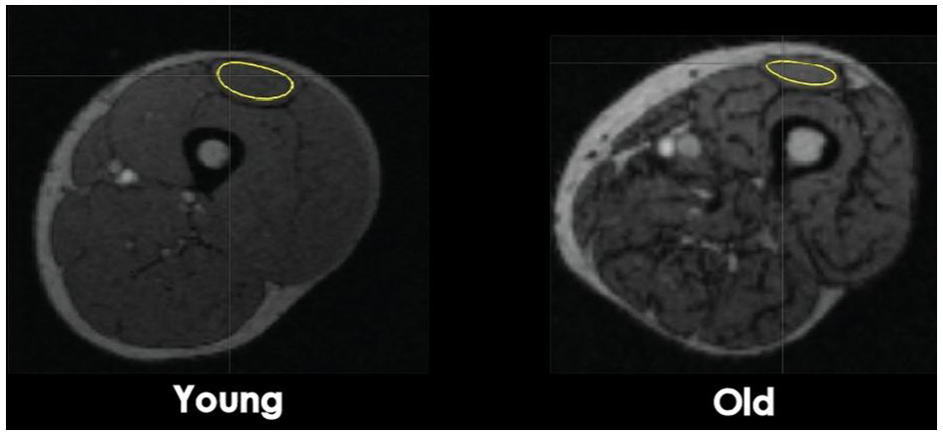
**Transaxial CT images at a mid-thigh level**. Computed Tomography images of a cross-section of the femoral region from a young and old man subject. The regions of interest (ROI) used for glucose uptake heterogeneity analysis were drawn around the quadriceps femoris (QF) muscles, four knee flexors muscles, and three hip muscles. For muscle volume analysis, ROIs were draw around the four next extensor muscles. ROI of the rectus femoris is shown as an example.

**Figure 2:**
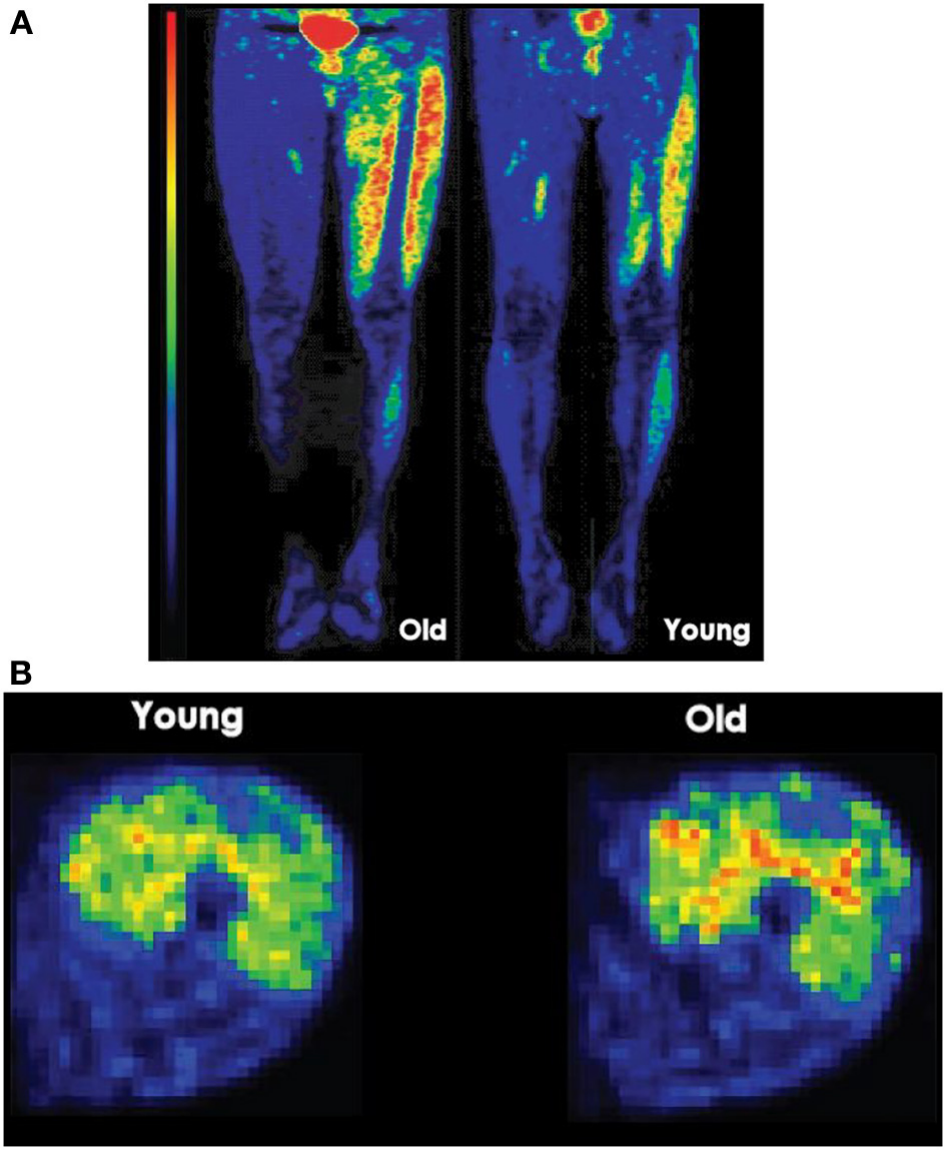
**(A)** Lower limb PET images after position and force tasks. Longitudinal PET images (ventral) taken after a young and an old man performed a position task for a similar duration. The high signal intensity (red) at the center of the pelvis resulted from accumulation of [^18^F]-FDG in the bladder. Red denotes the greatest signal intensity followed by yellow, green, and blue. **(B)** Glucose uptake heterogeneity in quadriceps femoris muscle during a task. Voxel-by-voxel map of relative glucose uptake in the exercising quadriceps femoris muscles of one subject. To calculate heterogeneity measure, coefficient of variation, the standard deviation of glucose uptake values in voxels of each quadriceps femoris (QF) muscle are divided by the mean glucose uptake in voxels of each QF muscle and multiplied by 100.

Eleven regions of interest (ROI) were identified in the skeletal muscles of the lower limb. In the thigh section, defined as 50% of the distance from the femoral head to the knee joint, ROIs were located for the knee extensors (vastus lateralis, vastus intermedius, vastus medialis, and rectus femoris) and flexors (biceps femoris short and long head, semimembranosus, and semitendinosus). In the hip region, which was 30 mm above the femoral head, ROIs were located for adductor magnus, sartorius, and gracilis. ROIs on the PET images were identified using cylinders with reference to the CT image (Figure 1). Because the PET images were acquired immediately after the fatiguing contractions, the GU values closely reflected the uptake of [^18^F]-FDG during the sustained contractions (Kemppainen et al., 2002) (Figure 2A). Standardized uptake values (SUV) were calculated for each muscle: SUV = [tissue radioactivity concentration/(injected dose/subject body mass)] (Sadato et al., 1998). The relative dispersion (standard deviation/mean × 100%) of GU values in PET image voxels within each ROI was calculated as an index of spatial GU heterogeneity within the muscles (Figure 2B). The data were analyzed with the software package Carimas™ (Cardiac Image Analysis System), developed at the Turku PET Centre and validated by Nesterov et al. (2009).

### Muscle volume

Using Analyze® 11.0 software package (Rochester, MN), CT images were uploaded and areas of bone, fat, muscle, and large blood vessels were segmented as previously described (Tanoli et al., 2004; Bashir et al., 2008). Operator-defined threshold signal intensities were assigned to each compartment and only muscle was included in the muscle volume. On the CT images, four knee extensor muscles were identified and the trace tool was used to draw ROIs along the borders of the rectus femoris, vastus lateralis, vastus medialis, and vastus intermedius on every transaxial slice for which these muscles were identifiable. Analyze software exported a muscle volume value, which is determined by multiplying cross sectional area by slice thickness and summing up all values for each muscle.

### Statistical analysis

The dependent variables included: time to failure for the position task, GUh values in the selected muscles, and muscle volumes. Assessments with the Kolmogorov-Smirnov test confirmed that the distributions were normal. A two-factor, repeated-measures ANOVA (task × age) was used to compare endurance times for the force and position tasks between young and old men. Changes in MVC force, and GUh were examined with a two-factor, repeated-measures ANOVA (task × age). A repeated-measures ANOVA was also performed to test the significance of differences in GUh between muscles. Two-factor, repeated-measures ANOVA were used to compare muscle volumes of left knee extensors (muscle × age) and physical activity levels (physical activity level × age) between young and old men. After a significant *F*-test, pairwise differences were identified using paired and unpaired *t*-test with Bonferroni corrections as *post-hoc* tests. Multiple linear regressions and the associated partial correlations (*r*) were performed to examine the contribution of each independent variable (muscle volume, physical activity levels, muscle strength) to GUh. The associated partial correlations (*r*) were used to identify the unique contribution of each independent variable to GUh and guided the stepwise selection procedure. The goodness of fit of the model, which indicates how well the linear combination of the predictor variables predicted the criterion variable, is reported as the squared multiple correlation (*R*^2^). The relative importance of the predictors was estimated with the partial correlations (*r*), which provide the correlation between the criterion variable and a predictor variable when the linear effects of the other independent variables in the model have been removed from both. A positive sign of the partial correlations indicates that the predictor and the criterion are positively related, whereas a negative sign indicates that they are inversely related. The significance level was set at *P* < 0.05. Statistical analyses were performed with SPSS software (SPSS version 17.0). Data are reported as means ± SD within text and tables and displayed as means ± s.e.m. in figures.

## Results

As reported previously (Rudroff et al., 2013), the MVC force at the beginning of each session was greater for the young men (462 ± 77 N) than for the old men (354 ± 91 N, *P* < 0.001). The target force for the two fatiguing contractions was 115 ± 19 N for the young men and 89 ± 20 N for the old men. There was no difference in endurance time between groups for the position task (943 ± 153 vs. 835 ± 92 s, *P* = 0.166). Accordingly, the young (848 ± 137 s) and old men (751 ± 83 s) performed the contractions prior to the PET/CT imaging for similar durations (*P* = 0.17). Moreover, the decline in MVC force immediately after the contractions was similar for young and old men (24.5 ± 7.5 and 22.9 ± 4.3% MVC, *P* = 0.247). However, MVC force was decreased to a greater extent after the position task had been performed to 90% of endurance time compared with the force task for the young men (28.8 ± 2.8 and 20.2 ± 7.7% MVC, *P* = 0.02) and for the old men (26.1 ± 2.6 and 19.6 ± 2.4% MVC, *P* = 0.017) Young men were more physically active than the old men (9193 ± 1829 and 4893 ± 2518 avg. steps/day, *P* = 0.004) (Rudroff et al., 2013). Muscle volumes of knee extensors were greater for the young men compared to the old men extensors (*P* = 0.004) (Table 1).

**Table 1:** **Muscle volumes (cm^3^) of the left knee extensors**.

	**Young men**	**Old men**
Knee extensors	1016 ± 163^**^	598 ± 70
Rectus femoris	183 ± 48^**^	107 ± 21
Vastus lateralis	255 ± 36^**^	146 ± 26
Vastus medialis	376 ± 102^*^	189 ± 54
Vastus intermedius	203 ± 67^*^	155 ± 33

*Muscle volumes (mean ± SD) for left knee extensor muscles of six young and six old men*.

* P < 0.05 and

** *P < 0.01 between young and old men*.

### GU uptake heterogeneity in skeletal muscles

Mean GU values have been presented previously and the reader interested on those is referred into this report (Rudroff et al., 2013). Plasma glucose concentration immediately prior to the infusion of the [^18^F]-FDG was similar for the young [88 ± 7 mg/dl (4.9 ± 0.4 mmol/l)] and old [92 ± 8 mg/dl (5.1 ± 0.4 mmol/l)] men, which ensured that the measurement of GU began from comparable baseline conditions for the two groups of participants. GUh was calculated for three-dimensional volumes of 11 leg muscles that were identified in CT images referenced to a standardized atlas (Table 2). There were no differences in GUh in knee extensors and flexors between force and position tasks for either group (task × muscle × age, *P* > 0.4); accordingly, data were collapsed across force and position tasks and compared between young and old men. The average GUh data (mean ± SD) for the eleven knee muscles were significantly greater for the old men (35.3 ± 3.3%) than for the young men (28.6 ± 2.4%) (age main effect, *P* = 0.007). Specifically, GUh was greater for the knee extensors (RF, VM, VL, and VIM) and one knee flexor (Bfs) of the old than the young men (muscle × age, *P* = 0.006).

**Table 2:** **Glucose uptake heterogeneity (%) in lower limb muscles after fatiguing contractions that required either force or position control**.

	**Force**		**Position**	
	**Young**	**Old**	**Young**	**Old**
Knee extensors	27.5 ± 4.1	35.1 ± 6.4^*^	25.6 ± 2.3	36.5 ± 6.5^*^
Vastus lateralis	26.6 ± 5.0	33.9 ± 6.3^*^	24.9 ± 2.0	35.8 ± 4.5^*^
Vastus intermedius	29.6 ± 5.3	39.2 ± 3.5^*^	25.9 ± 2.8	43.8 ± 3.9^*^^§^
Vastus medialis	28.5 ± 1.9	36.8 ± 5.7^*^	26.5 ± 2.2	35.5 ± 5.4^*^
Rectus femoris	25.2 ± 2.8	30.5 ± 5.6^*^	25.1 ± 2.4	30.8 ± 3.3^*^
Knee flexors	30.6 ± 3.1	35.6 ± 4.9^*^	30.8 ± 3.0	34.0 ± 5.2^*^
Biceps femoris short	31.4 ± 1.9	38.1 ± 5.2^*^	30.3 ± 2.4	37.7 ± 4.5^*^
Biceps femoris long	29.7 ± 2.6	36.8 ± 3.8^*^	31.0 ± 1.9	31.9 ± 3.1
Semimembranosus	31.0 ± 3.6	34.5 ± 5.2	31.9 ± 3.1	34.4 ± 4.7
Semitendinosus	30.2 ± 3.5	33.0 ± 3.0	30.2 ± 3.6	31.8 ± 5.5
Hip muscles	33.8 ± 5.0	33.4 ± 4.9	32.9 ± 5.3	39.5 ± 5.2^*^^†^
Adductor magnus	36.0 ± 2.6	33.0 ± 4.0	32.1 ± 3.7	38.7 ± 3.6^*^^†^
Sartorius	32.0 ± 5.6	33.0 ± 3.0	34.8 ± 5.3	40.0 ± 5.4^*^^†^
Gracilis	33.5 ± 5.0	33.0 ± 5.5	31.8 ± 5.7	40.1 ± 5.7^*^^†^

*Heterogeneity of GU uptake (mean ± SD) for left lower limb muscles of six young and six old men*.

* *P < 0.01 between young and old men*,

† *P < 0.01 between force and position tasks in old men*,

§ *P < 0.01 within leg extensors of old men*.

GUh of three hip muscles (AM, SA, GR) was similar between young and old men during the force task (33.8 ± 5 and 33.4 ± 4.8%, *P* = 0.832) but greater for the old men during the position task (32.9 ± 5.3 and 39.5 ± 5.2%, *P* < 0.001).

### Predictions of GU heterogeneity

**Figure 3:**
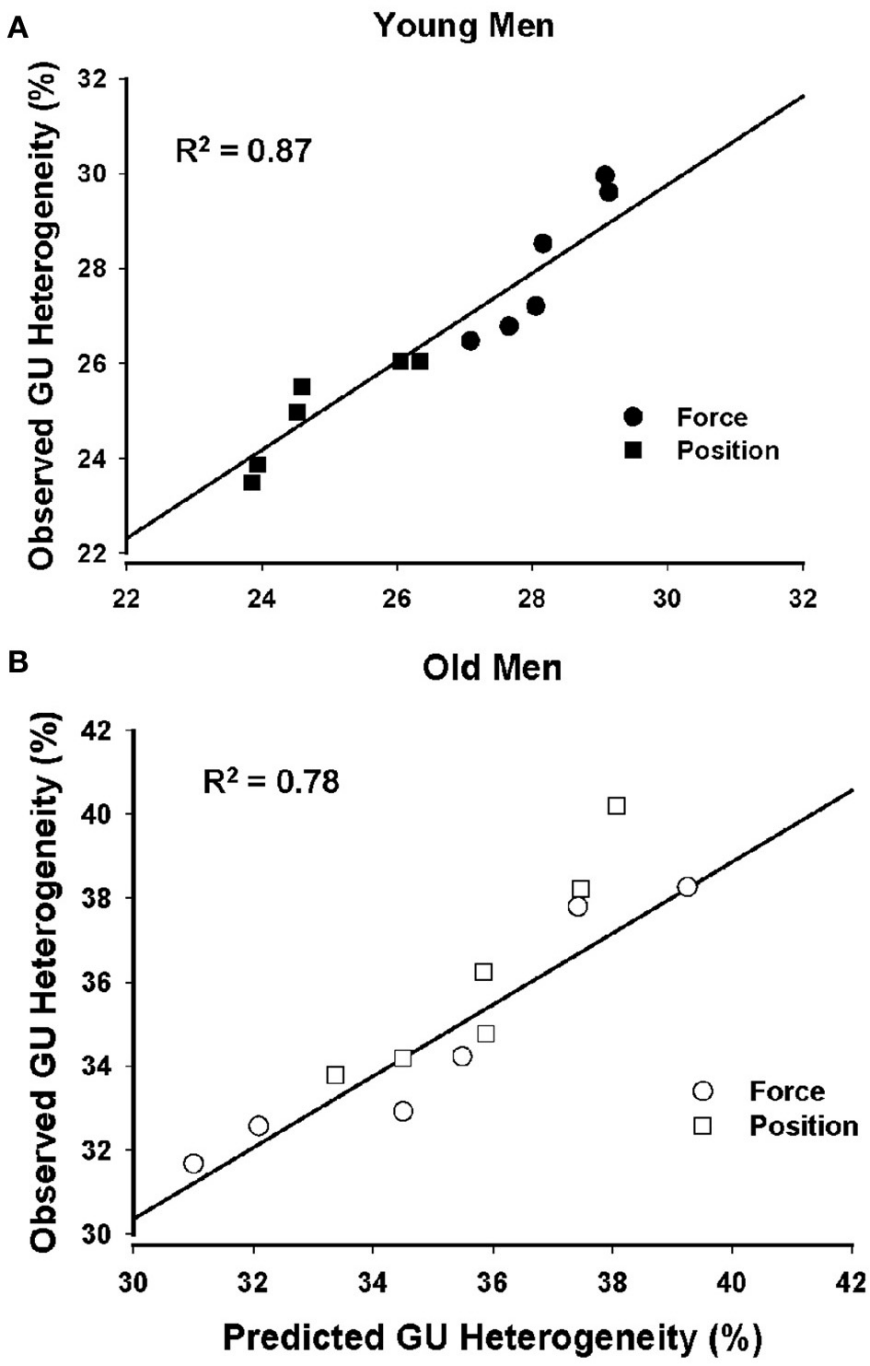
**Prediction of glucose uptake heterogeneity of young and old men**. Glucose uptake heterogeneity was strongly predicted by the MVC force of the knee extensor muscles for the young men **(A)** and by the muscle volumes of the knee extensors for the old men **(B)**.

Stepwise linear regression analysis using forward selection was adopted to use a parsimonious model using muscle volumes, physical activity levels, and MVC forces of young and old men to predict GUh. The stepwise procedure converged on a model for young men (*R*^2^ = 0.87; *P* < 0.001) that included MVC force (partial *r* = −0.95, *P* = 0.001) and indicates that young men with greater MVC force had less GUh (Figure 3A). Physical activity level (partial *r* = −0.02, *P* = 0.961) and muscle volume (partial *r* = −0.25, *P* = 0.462) were not associated with GUh. The model for old men (*R*^2^ = 0.78; *P* < 0.001) included muscle volume of the knee extensors (partial *r* = −0.87; *P* = 0.001) and indicates that old men with greater muscle volume had less GUh. Physical activity level (partial *r* = 0.13, *P* = 0.714) and MVC forces (partial *r* = 0.56; *P* = 0.126) (Figure 3B) were not associated with GUh.

## Discussion

The main finding of this study was that GUh as an indicator of heterogeneity in muscle activity was greater in knee muscles of old men compared with young men during two types of isometric contractions. For the old men, greater GUh was accompanied by smaller muscle volume, suggesting a role for muscle atrophy in the greater spatial variability of GU and muscle activity.

Our previous study indicates that old men used a strategy with higher metabolic costs (coactivation) during force and position tasks because of their inability to modulate muscle activation across tasks (Rudroff et al., 2013). Our previous findings of greater glucose standardized uptake values (SUV) for old men, together with our current findings of greater GU heterogeneity, suggest that old men employ different muscle activation strategies when performing the tasks that differ from those used by young men. This include both increased use of stabilizing muscles, as indicated by mean GU results in the previous study (Rudroff et al., 2013) and more heterogeneous activation of different muscle areas, as indicated in the present study.

Muscle force production is determined by the recruitment of motor units and their discharge rates. It is well known that many untrained old adults exhibit decreased strength and muscle mass, but the exact contributions of various underlying mechanisms are still unclear. A likely reason for impaired force production is a reduction in muscle fiber size and number associated with restructuring and subsequent loss of motor units, as well as reduced trophic stimulus from physical inactivity. Considering these age-related changes in muscle fiber morphology, we speculate that the old adults are not as able to effectively recruit more motor units during the isometric contractions and thus relied on the already recruited muscle fibers (motor units), resulting in greater GUh.

Accordingly, young men in this study were able to modulate motor unit (MU) activation and to recruit additional motor units, which were observed as less heterogeneously distributed GU in the muscle when compared to their old counterparts. This finding is in line with a previous study which used multi-channel surface EMG to compare spatial EMG potential distribution during force production between elderly and young men (Watanabe et al., 2012). They showed that the increase of heterogeneity and change of pattern in spatial EMG potential distribution with increase in exerted torque level are smaller in elderly men than in young men during submaximal isometric contractions in the vastus lateralis muscle. Specifically, the coefficient of variation of EMG amplitude stayed the same in elderly and increased in young men which indicated that old men recruited a limited number of new MUs whereas the young men altered MU activation.

Muscle volume of the knee extensors was significantly smaller for old than young men and was an important predictor of greater GUh for old men. This difference in muscle volume reflects the age-related morphological changes in the skeletal muscle, i.e., muscle fiber atrophy and decrease in number of fibers and motor units (Lexell et al., 1986; Sjostrom et al., 1986; Lexell and Downham, 1991; Deschenes, 2004; Lang et al., 2010). Morphological changes in the aging neuromuscular system include the shift to a less heterogeneous type of muscle tissue due to either a conversion from type II to type I muscle fibers or the preferential loss of type II muscle fibers (Petrofski and Lind, 1975; Larsson and Karlsson, 1978; Clarkson et al., 1981). Accordingly, GUh can be explained by spatial heterogeneity in the location of different types of muscle fibers (Chanaud and Macpherson, 1991) and a clustering of muscle fiber innervated by one motor unit in limited territory (Lexell and Downham, 1991). Furthermore, change in discharge rates of recruited MUs is also considered as one of the causes for change in spatial activation distribution during isometric contraction at a constant force level (Kleine et al., 2000; Holtermann and Roeleveld, 2006). Moreover, denervation of muscle fibers and/or increase of intramuscular fat tissue in may also induce greater GUh in old men (Buford et al., 2012).

Although physical activity levels and MVC forces were not significant predictors of GUh in the regression model for old men, highly trained old men can exhibit lower heterogeneity values. The old men in this study were less physically active and had lower MVC forces than the young men which suggest an impaired neural drive to muscles during MVCs in old adults (Enoka, 1997). Heinonen et al. (2012) investigated GUh during cycling at different intensities. GUh decreased with higher cycling intensity in some knee muscles but not in all which was explained by some muscles not activated sufficiently due to lower forces exerted by the subjects. It is suggested that lower GUh could be achieved by highly trained old men who could obtain and sustain higher target torques during the fatiguing contractions. For example, Pearson et al. (2002) compared master weight lifters (40–87 years) to age matched untrained controls and found that weight lifters had greater force production and muscle volumes compared to the controls. Previous studies have shown that greater lean leg volume (Harridge et al., 1999), relatively greater size and area of their type II fibers (Tesch et al., 1984; Larrson et al., 1997) might explain their superior muscle function. Future studies that emphasize studying master athletes are required to investigate the relation between aging, muscle disuse, and heterogeneity of activation.

GUh of the knee extensors and flexors did not differ between force and position task in either age group. One possible explanation might be that contraction intensity was too low to activate the muscles less heterogeneously which could be related to the supine posture and a more extended knee angle than in a previous study (Rudroff et al., 2010). However, GUh of three hip muscles (adductor magnus, sartorius, gracilis) was similar between young and old men during the force task but greater for the old men during the position task which further supports the explanation that old men in this study used accessory muscles rather than modulation of motor unit activity.

The use of PET scan after the exercise with ^18^F-FDG tracer is based on the tracer accumulation into the tissues where it has been taken up. ^18^F-FDG is taken up into the cells similarly as glucose, facilitated by GLUT4 transporter in skeletal muscle cells. After the entrance into the cells, most of the ^18^F-FDG is phosphorylated, but due to chemical properties, it cannot go further or back in metabolism, and it is trapped into the cells. In the present study, this method was used to estimate heterogeneity in muscle activity in PET image voxels during the exercise bouts. As the actual PET scan is performed after the exercise, a part of the accumulation is taking part also during the transition period into the scanner and during the actual scan. Previous calculations suggest that more than 90% of the tracer injected is taken up during the time of 25 min prior to the termination of dynamic whole-body exercise (Kemppainen et al., 2002). In our study the time from the tracer injection to termination of isometric exercise was less than that and exercise was also local for one leg. This suggests that less of the tracer was taken up during the actual exercise bouts. However, based on the fact that GLUT4 transporters remain in the cell membrane up to hours post exercise (Goodyear, 1998) and thereby facilitate GU, we have all the reasons to believe that post-exercise uptake of glucose was highly correlated with uptake during the exercise. Therefore, the measured GU heterogeneity reflects very well the heterogeneity in muscle metabolic activity during the exercise.

In conclusion, the findings of the current study demonstrate greater heterogeneity in GU in old men during two types of isometric contractions with the knee extensors. The GU measurements of muscle activation obtained with PET/CT imaging are consistent with age-associated differences in the modulation of muscle activation during tasks that require force or position control, but provide greater spatial information about the magnitude of the difference in muscle activity between young and old men when performing isometric contractions.

## Author contributions

Thorsten Rudroff contributed to (1) conception and design of the experiments; (2) collection, analysis and interpretation of data; and (3) drafting the article and revising it critically for important intellectual content. John H. Kindred and John-Michael Benson contributed to (1) analysis and interpretation of data; and (2) preparation of figures and tables. Brian L. Tracy contributed to (1) interpretation of data; and (2) drafting the article and revising it critically for important intellectual content. Kari K. Kalliokoski contributed to (1) conception and design of the experiments; (2) analysis and interpretation of data; and (3) drafting the article and revising it critically for important intellectual content. All authors approved the final version of the manuscript.

## Funding

The work was supported by an award (AG033744) from the National Institute on Aging (to Thorsten Rudroff).

### Conflict of interest statement

The authors declare that the research was conducted in the absence of any commercial or financial relationships that could be construed as a potential conflict of interest.
